# Our New Staff

**Published:** 1903-01

**Authors:** 


### EDITORIALS.

### OUR NEW STAFF.

During the year 1903 the editorial direction of the Journal will be enhanced by the assistance of the following members of the profession:

Dr. Leonard Pearson, Dean of the Veterinary Department of the University of Pennsylvania and State Veterinarian, will contribute to the editorial direction of the Journal.

Dr. M. E. Knowles, State Veterinarian for Montana, and one of the most keenly interested veterinarians in the great stock-growing industries of the West, will keep our readers informed of the progress of State sanitary control work.

Dr. John J. Repp, of the Veterinary Department of the Iowa Agricultural College and Secretary of the American Veterinary Medical Association, will conduct the Society Proceedings Department of the Journal and will add much to the interest in association work through the Journal's columns.

Dr. S. J. J. Harger, of the Veterinary Department of the University of Pennsylvania will continue to direct the Department of Foreign Abstracts, culling from the progress in Europe of comparative medicine as recorded in the journals of our profession and those of comparative medicine.

Dr. E. M. Ranck, Secretary of the Pennsylvania and Keystone Veterinary Associations, and for a number of years connected with the bacteriological laboratory of one of the chief firms of our country, will from time to time contribute the results of the most recent work at home and abroad.

The Senior Editor and Business Manager, Dr. W. Horace Hoskins, will continue to direct the general work of the Journal, and from time to time contribute interesting matter to the readers and the profession.

